# The Book World of Medicine and Science

**Published:** 1904-11-26

**Authors:** 


### The Book World of Medicine and Science.

Medical Monograph Series, No. 9: Adenoids. By Wyatt Wingrave, M.D. (London: Baillière, Tindall, and Cox. 1904. Pp. 128. 2s. 6d. net.)

This is a simple and straightforward account of adenoids, and is intended to be of service to students and practitioners. Dr. Wingrave gives excellent advice on when operation should be undertaken, but deals rather cursorily with palliative treatment, a subject on which advice is much needed, we think, at the present day. The author gives some useful hints on prognosis, pointing out that although adenoids may not be present when the child is first examined, yet they may subsequently give rise to symptoms and require operation. This is a contingency which it is wise to bear in mind. Again, symptoms may recur after operation, and if the patient's friends have not been warned of this possibility they will very likely assume that the operation has been faulty. The author might have also mentioned that adenoid vegetations, like tonsils, may show considerable variations in size from time to time. We can recommend this book to students and practitioners alike.

Serums, Vaccines, and Toxines in Treatment and Diagnosis. By W. Cecil Bosanquet, M D.Oxon., F.R C.P.Lond. (London: Cassell and Company. 1904. Pp. 344. Price 7s. 6d.)

Dr. Bosanquet is to be congratulated on the very good judgment with which he has selected from out of a large mass of somewhat confused material just the facts on which medical men are likely to desire information. The author sums up the main scope of the book in the preface where he states that he has endeavoured to give a short account of the principal facts which have been ascertained and of the general trend of speculation on the subject of immunity, as well as to elucidate the practical value of the many remedial agents which have been introduced in the form of serums, vaccines, and toxines. As a trustworthy account of the present position of serum-therapy the book will appeal to all practitioners who desire to keep themselves abreast of the times. The author has the advantage of a particularly lucid manner of expression which renders the book a very pleasant one to read.

Lectures on Diseases of Children. By Robert Hutchison, M.D., F.R.C.P. (London: Edward Arnold. 1904. Pp. 338. Illustrations 52. Price 6s.)

It is difficult in these days of much writing and many writers to find a gap in medical literature. We believe that the author of this volume has not only found one, but has succeeded in filling it in a most thorough manner. He has not written a complete text-book on the subject of diseases of children, but in addressing himself to his student audience he has selected with great skill those points which will prove useful to them in the diagnosis and treatment of children's ailments. Dr. Hutchison is a recognised authority on the subject of diet, and in the opening chapters he deals with that question. What strikes the reader is the simplicity of the methods, no elaborate percentage calculations, no laboratory treatment, but plain directions, which can be carried out in the ordinary domestic circle, as to the feeding of infants in health and in disease. This subject is so painfully elaborated and is wrapped up in such mystical ceremonial in the current text-books, that only a writer of established reputation would dare to treat the subject simply and in simple language. Chapters follow on various diseases in childhood which we cannot discuss in detail. The style of writing is dogmatic, but we are bound to confess the dogmatism is justified by its soundness. Not until the last chapter do we find any trace of faddism, but on the subject of adenoid vegetations in the nasopharynx we seem to recognise the traces of a hobby. The dogmatism disappears and an appalling list of diseases is given, which the author or someone else has found or thought he found, associated with adenoids. The best part of this chapter appears to us to be the warning to the students against the danger of exaggerating the importance of adenoids as a cause of remote disturbances. The book is fully illustrated from photographs, and these form a most instructive series of pictures of various diseased conditions. Much information can be conveyed by good illustrations, as, for instance, a picture of what the author calls "the syphilitic wig," and which is new to us as a symptom and as an illustration. We heartily recommend these lectures to the young practitioner or the senior student who wishes a medical guide to the pathological side of childhood.

The Student's Handbook of Surgical Operations. By Sir Frederick Treves, K.C.V.O. New Edition, revised by the author and Jonathan Hutchinson, jun., F.R.C.S. (London: Cassell and Company. Price 7s. 6d.)

As the name indicates, this work is intended to be of service to the student of medicine rather than to the qualified practitioner. It is an admirable handbook for the use of those who are carrying out an instructional course of operations on the dead body. The work, which is abridged from the larger "Manual of Operative Surgery," is sufficiently well known as a useful medical text-book.

Mediterranean Winter Resorts: A Complete and Practical Handbook to the Principal Health and Pleasure Resorts on the Shores of the Mediterranean. By E. A. Reynolds-Ball, F.R.G.S. 5th Ed. Pp. 618. (London: Hazell, Watson, and Viney, Ltd., 1904. Published in two parts, 3s. 6d. each, or in one volume, on India paper, 6s.)

What "Bædeker" is to the ordinary tourist "Reynolds-Ball" has become to the invalid and health seeker. The fifth edition of this invaluable guide contains much new material. The work has been considerably enlarged. Sections have been added dealing with such potential winter resorts as Cyprus and Kartoum, and fuller descriptions are given of some comparatively little-known winter places on the Levantine Riviera, and elsewhere. The hotel information appears to have been thoroughly revised and brought up-to-date. There is much in these pages that will be of particular service to English and American residents. We specially commend the many admirable articles on the principal invalid stations written by experienced resident English physicians. There is a very good map, and in the body of are a number the work there of useful diagrams. We consider this handbook a model one.

Notes on Assouan. By G. Dundas Edwards, M.A.Camb., M.R.C.S., L.R.C.P., L.S.A. Pp. 36. (London: John Bale, Sons, and Danielsson. 1904. Price 1s. net.)

Mr. Edwards in this brochure presents many meteorological and climatological considerations regarding the Nile district, and particularly Assouan, the distinctive features of which health resort are purity of air, large amount of sunshine, and warmth and dryness of the atmosphere. Good advice is given with regard to food, clothing, and exercise, and a special section is devoted to sun and sand baths. These "Notes" are written in a scientific spirit, and by one who has thought much and observed carefully. We commend them to the careful consideration of all health seekers who intend to visit the Land of the Pharaohs.

### THE BOOK WORLD OF MEDICINE AND SCIENCE—*Continued.*

The Nutrition of the Infant. By Ralph Vincent, M.D. Second Edition. (London: Ballière, Tindall and Cox. 1904. Demy 8vo. Pp. xx + 321. Price 10s. 6d. net.)

This work is a further contribution to the vast amount of medical writing which has been poured out so freely within recent years on the subject of the feeding of infants. One naturally inquires first of all what fresh light, if any, an author has to throw on this question. It would appear that he agrees with all medical writers in considering breast milk the best of all diets in early life, which is satisfactory, but he deplores that, in his hands, artificial feeding with cow's milk has proved a complete failure. In his despair he turned to America, and found there in the laboratory milk system of Dr. Rotch a method of feeding infants which has fulfilled all his disires. This work is an exposition of the American method of substitute feeding. As regards its applicability to this country some have ventured to entertain doubts. A working scheme with milk thus prepared demands a very special dairy farm, a number of expert chemists and mechanics to prepare the milk mixtures, a paterfamilias who is prepared to pay fivepence per pint instead of the ordinary twopence, and a physician who is at once a skilled physiologist, chemist, pathologist, clinician, and preferably a senior wrangler. We can only admire, at a great distance, the skill which is required in determining whether an infant should have ·60 or ·75 per cent. of caseinogen in his milk, whether the whey proteid should amount to as much as ·75 per cent., or a fatal effect be induced by increasing it to ·90 per cent. We are glad to think that authorities on children's diseases in this country do not hold the views of the author on the impossibility of rearing healthy children on the ordinary mixtures of cow's milk, and that so far little general support has been given to this empirical method borrowed from America. The other parts of this work deal in a thorough manner with dairy farms, and certain chapters are devoted to such diseases as intestinal ailments, rickets, and scurvy, which do not call for special comment.

The After-Treatment of Operations. By P. Lockhart Mummery, M.B., F.R.C S. (London: Baillière, Tindall and Cox, 1904. Second edition. Price 5s. net.)

The appearance of a second edition so soon after the first is evidence that Mr. Mummery's book has had the appreciation which it merited. But little alteration has been made from the former edition. The chapter on Shock and Collapse has been slightly modified in accordance with recent work on those conditions, and so also have been the chapters on abdominal surgery. As the book deals with a section of surgery which is quite inadequately treated in the text-books, it is likely to prove of service to anyone who is in active practice.

The Sterilisation of the Hands: a Bacteriological Inquiry. By Charles Leedham-Green, M.B., F.R.C S. (Birmingham: 1904. Cornish Brothers. Pp. 102. Price 2s. 6d. net.)

This book should be carefully read by every surgeon; it is the best exposition we have seen on the subject of hand sterilisation. Mr. Leedham-Green has approached the matter quite impartially, and although the nine pages of bibliography at the end of the book show that he has sojourned long in the mazes of medical literature, his conclusions are founded solely on ocular testimony derived from thorough experimental inquiry. Without entering into the details of Mr. Leedham-Green's methods, attention may be drawn to certain of his conclusions. For example, he finds that the aqueous solutions of carbolic acid, perchloride and biniodide of mercury, and other antiseptics are practically powerless to affect the micro-organisms on the hands, while alcohol far surpasses all other agents for reducing the septicity of the skin. Of all methods tested, the best results were obtained by scrubbing the hands with soap and very hot water, rubbing them with methylated spirit for three minutes, and then scrubbing them for a minute or two with 70 per cent. sublimate-alcohol (1 in 1,000), and finally rubbing the hands dry with a sterile cloth. We gladly commend the book to the notice of the medical profession.

Urine Examination Made Easy. By Thomas Carruthers, M.B., Ch.B. (London: J. and A. Churchill, 1904. Pp. 32. Price 1s. 6d. net.)

We do not know that Mr. Carruthers by the production of this little book has succeeded in making urine examination any easier than it was before. The book, which is intended for nurses, gives in a systematic manner the methods of preparing urine specimens, and of applying such simple tests as nurses may be expected to know. In his desire for brevity the author has allowed the composition to suffer, and this, we think, is the chief defect in the book, which however may be regarded as a reliable guide for nurses.

Health and Disease in Relation to Marriage and the Married State. Vol. I. Edited by Dr. Senator and Dr. Kaminer. Translated into English by J. Dulberg, M.D. (London: Rebman, Limited. 1904. Pp. 498. Price 30s.)

This is an earnest attempt to deal with marriage and its results from a purely scientific standpoint. The book consists of thirteen chapters by as many authors. The chief portion of the book is devoted to the effects of marriage on the female partner and on the children. As Professor Ewald says in his chapter on diseases of the organs of digestion in relation to marriage, "the gain which accrues to the organism from this union is undoubtedly . . . greater in the male sex, and the poor wife derives from the married state a plenteous harvest of ill-health." The ill-health is, of course, chiefly connected with pregnancy, child-bearing, and lactation. Hereditary dispositions and congenital diseases are very fully discussed, but to our mind by far the most useful chapters are the last seven, all of which are devoted to practical medicine and pathology. The chief fault has been to include too much, and this is especially so with the earlier chapters. Many of the details given might have been omitted with advantage. As a book of reference for many problems which are a little off the beaten track of medicine the present volume fills a distinct want.

